# *Helicobacter pylori* eradication for low-grade gastric mucosa-associated lymphoid tissue lymphoma is more successful in inducing remission in distal compared to proximal disease

**DOI:** 10.1038/sj.bjc.6603708

**Published:** 2007-04-03

**Authors:** J S Kim, S J Chung, Y S Choi, J H Cheon, C W Kim, S G Kim, H C Jung, I S Song

**Affiliations:** 1 Department of Internal Medicine, Liver Research Institute, Seoul National University College of Medicine, Seoul, Korea; 2 Department of Internal Medicine, Yonsei University College of Medicine, Seoul, Korea; 3 Department of Pathology, Seoul National University College of Medicine, Seoul, Korea

**Keywords:** mucosa-associated lymphoid tissue lymphoma, *Helicobacter pylori*, long-term outcome, tumour location

## Abstract

A series of studies has shown that *Helicobacter pylori* eradication induces remission in most patients with low-grade gastric mucosa-associated lymphoid tissue (MALT) lymphoma. However, there have been few reports about the effect of bacterial treatment on the gastric MALT lymphoma in Korea, a well-known *H. pylori* endemic area. A total of 111 *H. pylori*-infected patients were prospectively enrolled in Seoul National University Hospital and 99 among them were completely followed up according to our protocol. After *H. pylori* eradication, tumoural response was evaluated by endoscopy and histopathology every 2–3 months till complete remission (CR) and every 6 months after achieving CR. Median follow-up period was 41 months (range, 11–125 months). *Helicobacter pylori* was successfully eradicated in all 99 patients and CR was obtained in 84 (84.8%) of 99 patients. The median time to reach CR was 3 months and 94% of CR is in continuous complete remission. Five patients with CR relapsed after 10–22 months without the evidence of *H. pylori* reinfection. Cumulative recurrence rate was 2.3, 7.7 and 9.3% at 1, 2 and 3 years, respectively. Tumours were mainly located in distal stomach (67.7%) and tumours in distal stomach were associated with more favourable response than those in proximal stomach (*P*=0.001). Majority of patients with low-grade gastric MALT lymphoma treated by exclusive *H. pylori* eradication have a favourable long-term outcome, offering a real chance of cure. Tumour location could be a predictive factor for remission following *H. pylori* eradication.

Recently, *Helicobacter pylori* eradication is considered well-accepted initial therapy in cases of localised (stage I) low-grade gastric mucosa-associated lymphoid tissue (MALT) lymphoma, in which recently published series confirmed 62–95% complete remission (CR) rate (Neubauer *et al*, 1997; Savio *et al*, 2000; Montalban *et al*, 2001; Fischbach *et al*, 2004; Wundisch *et al*, 2005). The variation of CR rates may be due to the heterogeneity of previous studies, particularly as regards of study design and clinical characteristics. Data in some articles are lacking regarding *H. pylori* infection status and/or tumour extension and stage. Our study addressed these issues by strict study design, which comprised precise inclusion criteria, complete tumour staging and meticulous endoscopic follow-up.

In stage-I cases, the depth of invasion seems to be a predictive factor because previous studies have shown the evidence that tumours that invade muscularis propria or beyond are more likely not to respond to eradication therapy than those restricted to submucosa (Sackmann *et al*, 1997; Steinbach *et al*, 1999; Ruskone-Fourmestraux *et al*, 2001). However, clinical determination of the depth of invasion is not perfect without the evaluation of a surgical specimen. Therefore, there remains a need for alternative prognostic markers for stage-I gastric MALT lymphomas at diagnosis. The aim of this large prospective single-centre study was to determine the long-term outcome of exclusive *H. pylori* eradication therapy on low-grade stage-I gastric MALT lymphoma and to define the predictive factors of clinical response.

## PATIENTS AND METHODS

### Patients and diagnostic criteria

From January 1996 to December 2006, consecutive patients with *H. pylori-*positive stage-I (Lugano classification) (Rohatiner *et al*, 1994) low-grade gastric MALT lymphoma were prospectively enrolled in single centre (Seoul National University Hospital). The diagnosis of low-grade gastric MALT lymphoma was made according to the criteria of Isaacson and the recently published World Health Organization (WHO) classification of lymphoid neoplasms for extranodal marginal zone B-cell lymphoma of MALT type.

### Initial stage work-up and *H. pylori* eradication therapy

Complete staging work-up (de Jong *et al*, 1999; Raderer *et al*, 2000) consisted of the detailed physical examination, chest X-ray, abdomen CT, endoscopic ultrasonography (EUS) and bilateral bone marrow examination. Due to a lack of availability in early period, EUS could not be performed in 28 cases.

*H. pylori* infection status was determined by histologic examination and rapid urease test (CLO™, Delta West, Bentley, Western Australia). *Helicobacter pylori* status was considered to be positive if any of the two tests was positive. Only patients with *H. pylori-*positive and stage-I gastric MALT lymphoma were enrolled in the study. All patients were assigned to the 7-day course of omeprazole (20 mg b.i.d.), amoxicillin (1000 mg b.i.d.) and clarithromycin (500 mg b.i.d.). Second-line therapy consisted of a quadruple regimen containing omeprazole 20 mg b.i.d., tripotassium dicitrate bismuthate 300 mg q.i.d., metronidazole 500 mg t.i.d. and tetracycline 500 mg q.i.d. for 7 days.

### Remission evaluation following *H. pylori* eradication

Response was assessed according to the WHO criteria: CR was defined as no macroscopic tumour, histologically confirmed; a partial response when at least 50% tumour reduction was found; stable disease in case of variation within either 50% decrease of 25% increase; and progressive disease when an increase of at least 25% was present (Wotherspoon *et al*, 1993). Treatment was considered to have failed if patients showed no improvement or did not meet criteria for CR within 1 year after *H. pylori* eradication.

**Figure 1:**
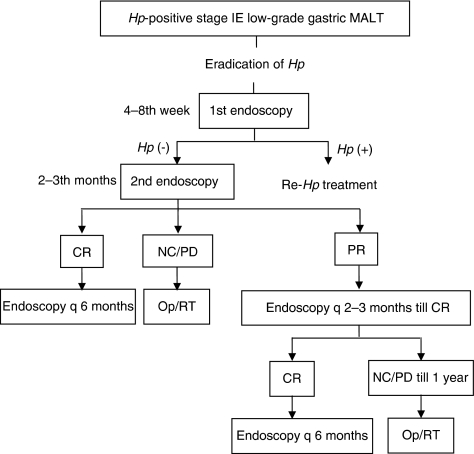
Schematic study design for the treatment approach and follow-up of patients with *H. pylori*-positive early-stage low-grade gastric MALT lymphoma. *Hp*=*Helicobacter pylori*; MALT=mucosa-associated lymphoid tissue; CR=complete remission; NC=no change; PR=partial response; Op=operation; RT=radiation therapy.

Patients first underwent follow-up endoscopy at 4–8 weeks after the completion of antibiotic therapy for the evaluation of *H. pylori* eradication. For determination of the clinical response, endoscopy with multiple biopsies from tumours and suspicious areas was performed at 2–3 months after the confirmation of eradication. Then follow-up endoscopy was repeated every 2–3 months until CR was achieved or until treatment failure for 1 year. In cases with CR, endoscopic examination and biopsy were performed every 6 months and the median number of endoscopies the patients in CR was nine. At each follow-up endoscopic examination, two separate biopsies from macroscopically normal mucosa of the antrum and body were evaluated for *H. pylori* infection. In cases of treatment failure to *H. pylori* eradication, patients were referred for surgery or radiation therapy (Figure 1).

### Statistical analysis

Data are expressed as median with range. In between-group comparisons, continuous variables were analysed by the Student's *t*-test and categorical variables using the *χ*^2^-test or Fisher's exact test. The Kaplan–Meier method was used to estimate remission duration. Analyses were performed using the Statistical Package for the Social Sciences (version 12.0; SPSS INC. Chicago, IL, USA). All statistical tests were two-sided, and a value of *P* <0.05 was considered to be statistically significant.

## RESULTS

### Clinicopathologic and endoscopic characteristics

A total of 111 patients were enrolled initially and 12 patients did not complete our protocol. Finally, 99 of the patients (M:F, 41:58; age median 52 years (24–77 years)) were evaluated in this study. There were fewer male than female patients and the male to female ratio was 1:1.4.

The macroscopic tumour type was determined according to the classification of Watanabe with minor modifications, as follows (Watanabe, 1980): (i) protruding, mass-like (9.1%); (ii) granular, gastritis-like (19.2%); (iii) depressed, erosion (50.5%); (iv) excavated, ulceration (15.2%); and (v) mixed (6.1%). The neoplasm was located in the distal stomach (antrum, angle and low body) in 67.7%. The depth of lymphoma involvement in the gastric wall was determined by EUS in 67 patients and by histologic examination of surgically resected specimen in 14 patients; most of the cases confined to the musoca (55.6%) or submucosa (34.6%) (Table 1).

### *H. pylori* eradication and tumoural response

Cure of *H. pylori* infection was documented in all patients (99 out of 99); eight patients needed a second-line treatment for successful eradication.

Median follow-up period was 41 months (11–125 months). Long-term outcome was characterised by CR of lymphoma in 84 out of 99 cases (84.8%). The median time to get CR after the completion of antibiotic therapy in *H. pylori*-eradicated patients was 3 months (1–20 months).

Table 2 shows the probabilities of CR stratified by clinicopathologic factors. The depth of tumour invasion determined by EUS or surgery strongly correlated with the outcome of eradication therapy. Complete remission was observed in 62 out of 73 (84.9%) patients with tumours that were limited to the mucosa or submucosa, but only in four out of eight (50.0%) patients with tumours that had invaded the muscularis propria or beyond (*P*=0.031). Interestingly, in our analysis, anatomic location in the stomach also influenced the tumour response. Patients with distal tumours had a higher CR rate (92.5%) than patients with proximal tumours (65.5%) (*P*=0.001). We analysed other clinical characteristics (age, sex, performance status, levels of haemoglobin, LDH and *β*2-microglobulin at diagnosis) that showed no statistically significant associations with the clinical response.

**Figure 2:**
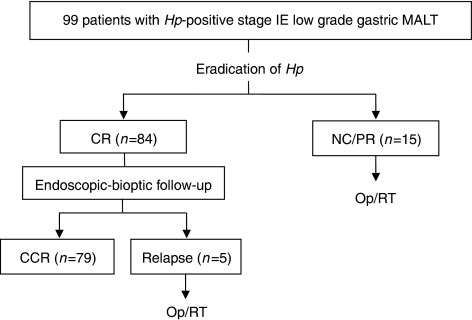
Clinical response and follow-up of the 99 patients on the basis of macroscopic and histologic findings. *Hp=Helicobacter pylori*; MALT=mucosa-associated lymphoid tissue; CR=complete remission; NC=no change; PR=partial response; CCR=continuous complete remission.

Fifteen patients (15.2%) showed persistent disease at 12 months after the eradication therapy. They underwent operation or radiation therapy and had CR in the subsequent follow-up (Figure 2).

### Recurrence of MALT lymphoma and reinfection of *H. pylori* during follow-up

**Figure 3:**
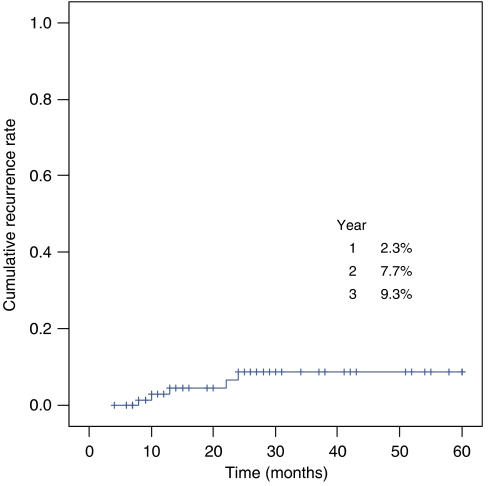
Recurrence of MALT lymphoma during a median 41 months follow-up period. Cumulative recurrence rate was plotted with Kaplan–Meyer survival analysis.

During median follow-up of 41 months, five patients (6.0%) relapsed with a low-grade lymphoma at 10–22 months after the remission without the evidence of reinfection; they were referred for alternative treatment (operation or radiotherapy) (Figure 2). Endoscopic appearances, location, depth of invasion and *H. pylori* status were not associated with recurrence (Table 3). Cumulative recurrence rate by Kaplan–Meyer survival analysis was 2.3, 7.7 and 9.3% at 1, 2 and 3 years after CR, respectively (Figure 3).

*H. pylori* has reinfected in 11 out of 99 patients (11.1%). But there was no evidence of disease relapse and *H. pylori* was eradicated in all of them.

## DISCUSSION

Our study showed predominant distal stomach location of gastric MALT lymphoma (67.7%). Although MALT lymphomas can occur in any region of the stomach, they located most commonly in the antrum and low body (Isaacson, 1996; Kim *et al*, 2001; Paik *et al*, 2002; Kahl, 2003), which reflects localisation of the highest concentrations in colonised individuals of *H. pylori* organisms and acquired lymphoid tissue (Wotherspoon, 1998). Interestingly, in our analysis, the patients with treatment failure showed a significantly greater frequency of proximal tumour location (*P*=0.001). Our possible explanation is that the pathophysiology of proximal MALT lymphoma differs from that of distal lymphoma; proximal lymphoma might also be associated with *H. pylori* infection or may be *H. pylori*-independent autoimmune gastritis, which is generally proximal in distribution (Dixon *et al*, 1996). Recent studies indicate that autoimmune gastritis may derive from *H. pylori* infection and suggest that antibiotic resistant tumours may be sustained by autoantigen-responsive T cells (Negrini *et al*, 1991; Greiner *et al*, 1994). Hence, the subset of *H. pylori*-eradicated MALT lymphomas in proximal stomach might be associated with autoimmune-related or autonomous tumours in patients with previous *H. pylori* gastritis, which do not respond to *H. pylori* eradiation (Steinbach *et al*, 1999; Ruskone-Fourmestraux *et al*, 2001). Our unpublished data for 27 patients with *H. pylori*-negative gastric MALT lymphoma revealed the predominant proximal location of lymphomas (62.9%), which suggests an important role of *H. pylori-*independent pathogenesis in case of proximal MALT lymphoma.

Although two large-scale German groups have shown the long-term outcome of gastric MALT lymphoma after *H. pylori* eradication (Fischbach *et al*, 2004; Wundisch *et al*, 2005), reports about predictive factors for clinical response after eradication therapy are rare. Our results might be valuable on the development that endocsopic tumour location could be used to derive a prognosis for remission following *H. pylori* eradication.

The disease recurrence rate has been reported to vary from 7 to 12.5% in patients who once achieved CR (Neubauer *et al*, 1997; Savio *et al*, 2000; Stolte *et al*, 2002; Fischbach *et al*, 2004). In the update of the multicentre study of Fischbach *et al* (2004) comprising 90 patients, 4.4% relapsed after a median follow-up of 44 months. In another Italian series of Savio *et al* (2000) involving 76 patients, 8% recurred during a median follow-up of 28 months. Our study showed that MALT lymphoma recurred in 6.0% of patients with a median follow-up of 41 months. Cumulative recurrence rate was 2.3, 7.7 and 9.3% at 1, 2 and 3 years after CR, respectively. Recurrences were found at 10–22 months after establishment of CR without the evidence of reinfection or transformation to high-grade lymphoma. Endoscopic appearance, location, depth of invasion and *H. pylori* status were not associated with the recurrence.

At present, it is not known whether any specific characteristic of the patient or of the lymphoma may predispose to a late relapse. With respect to both the rate of recurrence and *H. pylori* reinfection, Neubauer *et al* (1997) reported only one case of *H. pylori* reinfection among four cases of local recurrence. Fischbach *et al* (2004) also observed similar results of *H. pylori* reinfection, one patient reinfected among four patients of relapse. Therefore, is *H. pylori* probably not necessary for the lymphomas to relapse after the remission? At the beginning, the low-grade B-cell clone is still dependent on T-cell help (and thus susceptible to eradication therapy), whereas, later in process of clonal evolution, *H. pylori* infection and T-cell help may not be necessary (Hussell *et al*, 1993, 1996). The relapses may thus indicate that the patients had B-cell lymphomas that had evolved from B-cell clones that were already further progressed when the cure of *H. pylori* infection had been performed. These data suggest that some relapses are a result of the development of a lymphomatous clone that is independent of the *H. pylori*-mediated antigenic drive.

In conclusion, *H. pylori* eradication as single therapy may be appropriate for early-stage gastric MALT lymphoma, which leads to a favourable long-term outcome, offering a real chance of cure. In addition to depth of invasion, our results highlight the relevance of the location of lesions as a predictive factor for tumoural response. Until the clinical and the cellular characteristics of *H. pylori*-dependent and *H. pylori*-independent tumours are better defined, it would be prudent to pursue a cautious approach to tailor the therapeutic strategy and follow-up of these patients.

## Figures and Tables

**Table 1:** Clinical and endoscopic characteristics (*n*=99)

**Clinicopathologic characteristics**	**No. of patients**	**%**
Age, median (range), years	52 (24–77)	
Sex (male:female)	41:58	
		
*Endoscopic appearance*		
I: protruding, mass	9	9.1
II: granular, gastritis	19	19.2
III: depressed, erosion	50	50.5
IV: excavated, ulceration	15	15.2
V: mixed	6	6.1
		
*Endoscopic location*		
Proximal^a^	29	29.3
Distal^b^	67	67.7
Multifocal	3	3.0
		
*Depth of gastric wall involvement* c		
Mucosa	45/81	55.6
Submucosa	28/81	34.6
Muscularis propria or beyond	8/81	9.9

a Mid body, high body, fundus or cardia.

b Antrum, angle or low body.

c Evaluated by EUS in 67 patients and by histologic examination of surgical specimen in 14 patients.

**Table 2:** Risk factor analysis for non-response of MALT lymphoma

**Characteristics**	**Non-response (*n*=15)**	**Response (*n*=84)**	***P*-value**
*Endoscopic appearance* (%)			NS
I: protruding, mass	3 (20.0)	6 (7.1)	
II: granular, gastritis	1 (6.7)	18 (21.4)	
III: depressed, erosion	6 (40.0)	44 (52.4)	
IV: excavated, ulceration	4 (26.7)	11 (13.1)	
V: mixed	1 (6.7)	5 (6.0)	
			
*Endoscopic location* (%)			0.001
Proximal^a^	10 (80.0)	19 (22.6)	
Distal^b^	5 (20.0)	62 (73.8)	
Multifocal	0 (0)	3 (3.6)	
			
*Depth of gastric wall involvement*^c^ (%)			
Mucosa	5/15 (33.3)	40/66 (60.6)	
Submucosa	6/15 (40.0)	22/66 (33.3)	0.064
Muscularis propria or beyond	4/15 (26.7)	4/66 (6.0)	0.031

MALT=mucosa-associated lymphoid tissue.

a Mid body, high body, fundus or cardia.

b Antrum, angle or low body.

c Evaluated by EUS in 67 patients and by histologic examination of surgical specimen in 14 patients.

**Table 3:** Risk factor analysis for recurrence of MALT lymphoma

**Characteristics**	**Recurrence (*n*=5)**	**No recurrence (*n*=79)**	***P*-value**
*Endoscopic appearance* (%)			NS
I: protruding, mass	0 (0)	6 (7.6)	
II: granular, gastritis	0 (0)	18 (22.8)	
III: depressed, erosion	4 (80.0)	40 (50.6)	
IV: excavated, ulceration	1 (20.0)	10 (12.7)	
V: mixed	0 (0)	5 (6.3)	
			
*Endoscopic location* (%)			NS
Proximal^a^	2 (40.0)	16 (20.3%)	
Distal^b^	3 (60.0)	60 (75.9%)	
Multifocal	0 (0)	3 (3.8%)	
			
*Depth of gastric wall involvement*^c^ (%)			NS
Mucosa	3/5 (60.0)	45/75 (60.0)	
Submucosa	2/5 (40.0)	25/75 (33.3)	
Muscularis propria or beyond	0 (0)	5/75 (6.7)	
			
Median time to CR (months)	3	3	NS
*H. pylori* reinfection (%)	0 (0)	11 (13.9)	NS

MALT=mucosa-associated lymphoid tissue.

a Mid body, high body, fundus or cardia.

b Antrum, angle or low body.

c Evaluated by EUS in 66 patients and by histologic examination of surgical specimen in 14 patients.
